# Association of a pro-inflammatory diet with type 2 diabetes and hypertension: results from the Ravansar non-communicable diseases cohort study

**DOI:** 10.1186/s13690-022-00839-w

**Published:** 2022-03-31

**Authors:** Samira Arbabi Jam, Shahab Rezaeian, Farid Najafi, Behrooz Hamzeh, Ebrahim Shakiba, Mehdi Moradinazar, Mitra Darbandi, Fatemeh Hichi, Sareh Eghtesad, Yahya Pasdar

**Affiliations:** 1 Student Research Committee, Kermanshah University of Medical Sciences, Kermanshah, Iran; 2 Research Center for Environmental Determinants of Health (RCEDH), Health Institute, Kermanshah University of Medical Sciences, Kermanshah, Iran; 3 Infectious Diseases Research Center, Kermanshah University of Medical Sciences, Kermanshah, Iran; 4 Cardiovascular Research Center, Kermanshah University of Medical Sciences, Kermanshah, Iran; 5 Behavioural Diseases Research Center, Kermanshah University of Medical Sciences, Kermanshah, Iran; 6 Internal Medicine Department, School of Medicine, Kermanshah University of Medical Sciences, Kermanshah, Iran; 7 Liver and Pancreatobiliary Research Center, Digestive Diseases Research Institute, Tehran University of Medical Sciences, Tehran, Iran; 8 Isar Square, School of Nutritional Sciences and Food Technology, Nutritional Sciences Department, Tehran, Iran

**Keywords:** Dietary inflammatory index, Pro-inflammatory diet, Hypertension, Type 2 diabetes, Persian

## Abstract

**Background:**

Most non-communicable diseases (NCDs) are associated to diet and inflammation. The Dietary Inflammatory Index (DII) is a developed and validated self-assessment tool. The study was conducted to assess the association of DII with the hypertension (HTN) and type 2 diabetes mellitus (T2DM).

**Methods:**

This cross-sectional analysis was conducted on 9811 participants aged 35 to 65 years from the Ravansar Non-Communicable Diseases (RaNCD) cohort study’s baseline phase data. The DII was calculated using 31 food frequency questionnaire parameters (FFQ). Univariable and multiple logistic regression was used to derive the estimates.

**Results:**

In healthy participants, the mean DII score was − 2.32 ± 1.60; in participants with T2DM, HTN, or T2DM&HTN, the mean DII score was − 2.23 ± 1.59, − 2.45 ± 1.60 and − 2.25 ± 1.60, respectively (*P* = 0.011). Males had a significantly higher pro-inflammatory diet than females (*P* <  0.001). BMI (body mass index), triglyceride, energy intake, smokers were significantly higher and socio-economic status (SES), physical activity and HDL-C were significantly lower in the most pro-inflammatory diet compared to the most anti-inflammatory diet. Participants with T2DM, HTN, and T2DM&HTN had significantly higher mean anthropometry indices (*P* <  0.001) and lipid profiles than healthy subjects (*P* <  0.001). After adjusting for age, gender, and physical activity, the probability of developing T2DM was 1.48 (95% CI: 1.19, 1.85) times greater in the fourth quartile of DII than in the first quartile.

**Conclusions:**

The findings of this study showed that an anti-inflammatory diet are associated with HTN, T2DM, and the risk factors associated with these conditions. Modification of diet is recommended to reduce inflammation.

## Background

Chronic systemic inflammation plays a critical role in the pathogenesis of non-communicable diseases (NCDs), such as cardiovascular disease (CVDs), type 2 diabetes mellitus (T2DM), metabolic syndrome (MetS), Hypertension (HTN), fatty liver, and cancer [1–5]. In type 2 diabetes and HTN, obesity and overweight, insulin resistance, and overexpression of pro-inflammatory proteins including C-reactive protein (CRP) and cytokines (IL-1β, IL-6, and TNF-α) contribute to chronic inflammation [6]. Additionally, environmental, psychosocial, and behavioural factors can lead to inflammation during times of stress [7], where inflammation can be reduced by modifying some of these factors. A diet is a vital part of the human lifestyle, and its modification can help moderate the inflammatory process [8, 9].

Previous research has demonstrated that certain dietary patterns, such as a high fibre, fruit, and vegetable intake and a low fat intake, reduce inflammatory markers and thus the risk of NCDs [10, 11]. Conversely, the Western diet (including processed meats and refined carbohydrates) is associated with elevated inflammatory markers and appears to be a risk factor for certain NCDs [12].

Various foods and food components can affect inflammatory markers in the blood. Recently, a relatively new index called the dietary inflammatory index (DII) had been used to assess the inflammatory potential of an individual’s diet. The DII is based on 45 food parameters. DII measures anti- to pro-inflammatory compounds, and a high value indicates that a person’s diet is inflammatory [13]. Given the diversity of lifestyles and dietary patterns found across geographical regions and ethnic groups, assessing the DII in diverse populations is critical. Given the rising prevalence of NCDs, prevention and control are essential. As a result, identifying and modifying modifiable risk factors for NCDs can help to reduce the disease burden in communities.

According to reports, HTN and its complications account for approximately 9.4 million deaths worldwide each year [14], with Asia being the region with the highest rate of type 2 diabetes [15]. As a result, data from the Ravansar non-communicable diseases cohort study (RaNCD), the only Kurdish cohort study currently being conducted, consists of approximately 10,000 adults. The aim of this study was to determine whether a pro-inflammatory diet as measured by DII was associated with an increased odd of HTN and T2DM in a Kurdish population.

## Methods

### Study setting and design

**Fig. 1:**
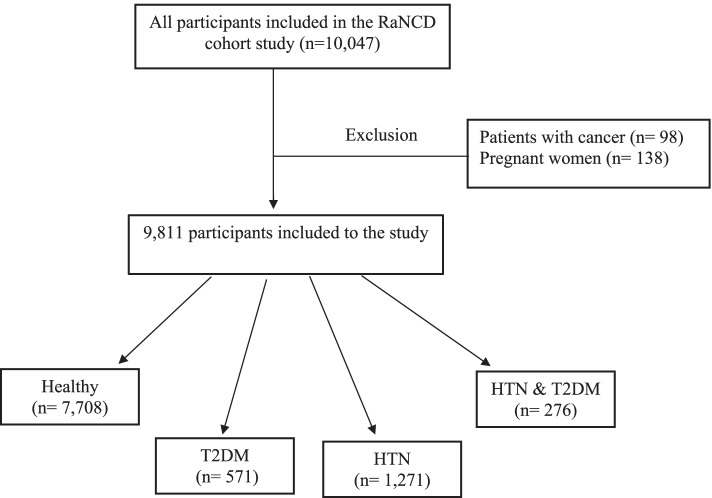
Flow chart of the cross-sectional study in the RaNCD cohort

We used baseline data from RaNCD in this cross-sectional study, one of the sub-studies of the national Prospective Epidemiological Research Studies of IrAN (PERSIAN) cohort study [16]. Ravansar is one of Kermanshah Province’s western cities, with a population of approximately 50,000 in western Iran. Comprehensive information about the RaNCD study has been published and is accessible [17]. This study enrolled participants aged 35–65 years’ old who were in the bassline phase of RaNCD (10,000 individuals). Due to the research design, subjects with cancer and pregnant women were excluded. The final population of the study consisted of 9811 adults (Fig. 1).

### Data collection

Sociodemographic data, such as age, gender, marital status, and residence location, as well as personal habits (smoking status and alcohol consumption), were collected face to face using digital questionnaires.

The socioeconomic status (SES) was determined using 18 items (housing, car price, dishwasher, freezer, washing machine, computer, laptop, internet access, motorcycle, color TV, TV type, bathroom, cell phone, vacuum cleaner, area per capita, room per capita, education level, and residence place); finally, the SES was classified into five groups from poorest to richest using the principal component analysis (PCA) method [18].

A standardized cohort study using a physical activity questionnaire (Including 22 questions) was used to assess participants’ physical activity on a met/hour per week basis. Participants were divided into three groups (light, moderate, high).

Blood samples were collected after 12 h of fasting to measure biochemical markers such as triglyceride (TG), low-density lipoprotein cholesterol (LDL-C), high-density lipoprotein cholesterol (HDL-C), and total cholesterol (T-C), as well as fasting blood sugar (FBS). A BSM 370 (with 0.1 cm precision) was used to measure the height (Biospace Co, Seoul, Korea).

Weight and other anthropometric indices, including body mass index (BMI), body fat mass (BFM), and visceral fat area (VFA), were measured using a Bio-Impedance Analyzer BIA (with a precision of 0.5 kg) (InBody 770 Biospace, Korea). Standard methods were used to determine waist circumference (WC) and waist to hip ratio (WHR). Blood pressure (BP) was measured via a manometer cuff and stethoscope after resting the arm in the seated position for 10 min.

### Assessment of the dietary inflammatory index

Food Frequency Questionnaire (FFQ) items were used to calculate the DII scores. Participants responded to questions about their consumption of various food groups in terms of quantity and frequency. They were shown a photo from the booklet to assist them in estimating portion sizes. At this stage, diet-related data were collected face-to-face to minimize measurement bias.

Shivappa et al. found that 45 foods were associated with one or more of the inflammatory markers Interleukin-1b (IL-1b), Interleukin-6 (IL-6), Tumor Necrosis Factor-α (TNF-α), C-reactive protein (CRP), anti-inflammatory markers Interleukin-4 (IL-4) and Interleukin-10 (IL-10). Z-scores for each parameter were determined through the method developed by Shivappa et al., using the mean and standard deviation (SD) of global intake. The Z-score was then converted to a percentile. This method was utilized to calculate the inflammatory score for each food parameter, and then the inflammatory scores for all parameters were added to obtain the total DII score. The higher the DII score, the more pro-inflammatory the diet, and the lower the DII score, the more anti-inflammatory the diet [19, 20]. DII scores were classified into four groups (quartiles) to assess associations. The first and fourth quartiles had the lowest and highest DII scores, respectively.

In the current study, DII was calculated using 31 food parameters, including carbohydrate, protein, total fat, trans fat, monounsaturated fatty acids (MUFA), polyunsaturated fatty acids (PUFA), cholesterol, saturated fat, omega-3, omega-6, vitamins A, B6, B12, C, D, E, selenium, zinc, energy, iron, magnesium, niacin, riboflavin, thiamine, beta-carotene, fibre, folic acid, caffeine, garlic, onion, and tea.

### Hypertension and type 2 diabetes mellitus assessment

HTN was defined as a systolic blood pressure (SBP) of ≥140 mmHg either-or a diastolic blood pressure (DBP) of ≥90 mmHg either-or as being currently on antihypertensive medication [21]. T2DM was defined as having an FBS (fasting blood sugar) of ≥126 mg/dl either-or being on diabetes medication either-or having diabetes confirmed by a health practitioner [22].

### Statistical analysis

The mean ± standard deviation was used for quantitative variables, while the frequency (percentage) was used via DII quartiles for qualitative variables. Additionally, Chi square test and one-way ANOVA compared the frequency (%) and mean ± standard deviation of basic characteristics among the quartiles of DII. Also, one-way ANOVA compared the mean ± standard deviation of anthropometric and biochemical characteristics among the four studied groups (Healthy, T2DM, HTN, T2DM & HTN). Crude and adjusted logistic regression models (Adjusted for potential confounders including age, sex, BMI, BFM, WHR, carbohydrate (%E), protein (%E) and oil/fat (%E) and physical activity) were used to determine the association between DII and hypertension and T2DM. The crude and adjusted odds ratios with 95% confidence interval were reported. *P* values < 0.05 were considered significant. All analyses were done with STATA software version 14.2 (Stata Corp, College Station, Tex).

## Results

### Basic characteristics

The current study entered 9811 participants aged 35–65 years. The baseline characteristics of the study participants according to DII quartiles are summarized in Table 1. Males consumed significantly more pro-inflammatory foods than females (*P* <  0.001). According to DII score quartiles, subjects with the most pro-inflammatory diets had a significantly higher BMI, higher TG, were heavy smokers, had a lower SES, engaged in less physical activity, and had a lower HDL-C. The DII score was significantly higher in urban residents (*P* <  0.001) and married subjects (*P* <  0.001). Increases in DII scores were associated with an increase in the prevalence of T2DM (*P* = 0.086) and HTN (*P* = 0.003). Daily energy intake (*P* <  0.001) and energy from lipids intake (*P* <  0.001) were significantly higher in subjects on a pro-inflammatory diet than in subjects on an anti-inflammatory diet.

**Table 1:** Baseline characteristics of the participants according to DII Quartiles (RaNCD cohort data, *N* = 9811)

Variable	Frequency (%), Mean ± SD				*P* value*
	Q1 (Most Anti-Inflammatory)	Q2	Q3	Q4 (Most Pro-Inflammatory)	
Frequency	*n* = 2451	*n* = 2430	*n* = 2463	*n* = 2467	
DII range	(−6.18, −3.54)	(− 3.54, − 2.68)	(− 2.69, − 1.38)	(−1.38, 4.27)	
Sex					< 0.001
Male	983 (40.11)	1083 (44.57)	1214 (49.29)	1449 (58.74)	
Female	1468 (59.89)	1347 (55.43)	1249 (50.71)	1018 (41.26)	< 0.001
Residence place					
Urban	934 (38.11)	1280 (52.67)	1674 (67.97)	1991 (80.71)	< 0.001
Rural	1517 (61.89)	1150 (47.33)	789 (32.03)	476 (19.29)	
Alcohol use					
No	2373 (96.82)	2334 (96.05)	2344 (95.17)	2278 (92.34)	< 0.001
Yes	78 (3.18)	96 (3.95)	119 (4.83)	189 (7.66)	
Socio-economic status					
1(poorest)	780 (31.84)	499 (20.54)	326 (13.24)	329 (13.35)	< 0.001
2	544 (22.20)	502 (20.67)	470 (19.08)	433 (17.57)	
3	410 (16.73)	510 (21)	519 (21.07)	525 (21.30)	
4	365 (14.90)	475 (19.56)	565 (22.94)	566 (22.96)	
5 (richest)	351 (14.33)	443 (18.24)	583 (23.67)	612 (24.83)	
Marital status					
Married	2114 (86.25)	2193 (90.25)	2245 (91.15)	2294 (92.99)	< 0.001
Single	156 (6.36)	105 (4.32)	90 (3.65)	67 (2.72)	
Widowed/Divorced	181 (7.39)	132 (5.43)	128 (5.2)	106 (4.3)	
Physical activity (Met-h/week)					
Light	722 (29.46)	788 (32.43)	749 (30.41)	716 (29.02)	< 0.001
Moderate	1242 (50.67)	1138 (46.83)	1233 (50.06)	1135 (46.01)	
High	487 (19.87)	504 (20.74)	481 (19.53)	616 (24.97)	
Smoking status					
No	1962 (80.08)	1962 (80.81)	1960 (79.61)	1892 (76.75)	0.003
Yes	488 (19.92)	466 (19.19)	502 (20.39)	573 (23.25)	
Diabetes					
No	2241 (91.88)	2221 (91.81)	2246 (91.56)	2209 (90.09)	0.086
Yes	198 (8.12)	198 (8.19)	207 (8.44)	243 (9.91)	
Hypertension					
No	2007 (81.88)	2073 (85.31)	2090 (84.86)	2095 (84.92)	0.003
Yes	444 (18.12)	357 (14.69)	373 (15.14)	372 (15.08)	
Age (year)	48.71 ± 8.46	47.58 ± 8.33	46.81 ± 8.16	46.20 ± 7.89	0.004
BMI (kg/m^2^)	26.99 ± 4.68	27.40 ± 4.62	27.58 ± 4.60	27.95 ± 4.56	0.668
WHR	0.94 ± 0.06	0.94 ± 0.061	0.94 ± 0.06	0.95 ± 0.06	0.054
TG (mg/dl)	134.20 ± 81.09	134.51 ± 76.98	137.99 ± 82.40	142.95 ± 89.37	< 0.001
HDL-C (mg/dl)	48.03 ± 11.55	46.94 ± 11.31	45.80 ± 11.31	44.49 ± 10.77	< 0.001
LDL-C (mg/dl)	103.40 ± 25.99	102.24 ± 25.23	101.18 ± 24.62	101.12 ± 25.73	0.041
T-C (mg/dl)	187.40 ± 38.69	185.95 ± 37.48	184.06 ± 36.85	183.63 ± 38.20	0.083
Energy intake (kcal/d)	1904.96 ± 691.66	2052.14 ± 720.88	2328.54 ± 822.37	2904.19 ± 1059.81	< 0.001
Carbohydrate (%E)	66.16 ± 8.69	65.37 ± 7.59	63.89 ± 7.48	62.63 ± 7.95	< 0.001
Protein (%E)	14.97 ± 1.73	15.58 ± 1.95	15.83 ± 1.94	16.56 ± 2.32	< 0.001
Oil/Fat (%E)	18.22 ± 8.47	18.61 ± 7.16	20.08 ± 7.14	21.03 ± 7.44	< 0.001

^*^P-value was obtained one-way ANOVA and Chi square test

The anthropometric and biochemical characteristics of the participants are listed in Table 2. The mean age was significantly higher in subjects with T2DM, HTN, and comorbidity (T2DM&HTN) than in healthy subjects (*P* < 0.001). Subjects with T2DM, HTN, and T2DM&HTN had significantly higher mean BMI, WHR, WC, BFM, and VFA than healthy subjects (*P* < 0.001). In subjects with T2DM, HTN, and T2DM&HTN, the mean lipid profile (LDL-C, TG, and T-C) was significantly higher HTN (*P* < 0.001).

**Table 2:** Anthropometric and biochemical characteristics of participant (RaNCD cohort data, *N* = 9811)

Parameters	Healthy (*n* = 7708)	Diabetes (*n* = 571)	HTN (*n* = 1271)	Diabetes & HTN (*n* = 276)	*P* value*
Age (year)	45.93 ± 7.88	50.44 ± 7.43	52.83 ± 7.72	54.22 ± 6.85	< 0.001
BMI (kg/m^2^)	27.17 ± 4.60	28.88 ± 4.35	28.48 ± 4.68	29.04 ± 4.45	< 0.001
WHR	0.94 ± 0.06	0.96 ± 0.06	0.96 ± 0.06	0.97 ± 0.06	< 0.001
WC (cm)	96.45 ± 10.40	100.58 ± 9.80	99.74 ± 10.67	101.54 ± 9.84	< 0.001
BFM (kg)	24.38 ± 9.47	27.82 ± 9.25	27.20 ± 9.57	28.42 ± 9.25	< 0.001
VFA (cm^2^)	117.93 ± 51.02	137.82 ± 49.26	135.55 ± 51.18	143.18 ± 49.65	< 0.001
DII	−2.32 ± 1.60	−2.23 ± 1.59	− 2.45 ± 1.60	−2.25 ± 1.60	0.011
TG (mg/dl)	131.03 ± 74.52	184.70 ± 128.79	147.56 ± 89.35	170.32 ± 98.01	< 0.001
HDL-C (mg/dl)	46.60 ± 11.35	43.78 ± 11.25	46.16 ± 11.08	44.30 ± 10.69	< 0.001
LDL-C (mg/dl)	101.31 ± 25.05	104.71 ± 27.73	104.68 ± 25.42	102.33 ± 29.04	< 0.001
T-C (mg/dl)	183.96 ± 36.96	191.74 ± 43.68	189.64 ± 38.15	187.44 ± 44.18	< 0.001
Energy intake (kcal/d)	2328.83 ± 918.87	2285.62 ± 906.03	2146.12 ± 904.61	2198.77 ± 976.80	< 0.001
Carbohydrate (%E)	64.44 ± 8.10	64.05 ± 8.05	64.65 ± 7.89	64.65 ± 7.89	0.021
Protein (%E)	15.66 ± 20.3	16.06 ± 2.16	15.94 ± 2.23	16.28 ± 2.27	< 0.001
Oil/Fat (%E)	19.60 ± 7.72	19.80 ± 7.52	18.76 ± 7.40	19.04 ± 7.10	0.002

^*^P-value was obtained one-way ANOVA

### Association between dietary inflammatory index and T2DM

The crude logistic regression model revealed that the probability of developing T2DM was 1.24 (95% CI: 1.02, 1.51) times greater in subjects with the most pro-inflammatory diet than in subjects with the most anti-inflammatory diet. After adjusting for age and gender, the odds of developing T2DM were 1.54 (95% CI: 1.26, 1.90) times greater in the fourth quartile of DII than in the first quartile (Table 3).

**Table 3:** Logistic regression analysis of association between dietary inflammatory index and T2DM

Diabetes	Dietary Inflammatory Index			
	Q 1 (Most Anti-Inflammatory)	Q 2	Q 3	Q 4 (Most Pro-Inflammatory)
	OR	OR (95% CI)	OR (95% CI)	OR (95% CI)
Model I	1	1.01 (0.82, 1.24)	1.04 (0.85, 1.28)	1.24 (1.02, 1.51)
Model II	1	1.10 (0.89, 1.36)	1.22 (0.99, 1.50)	1.54 (1.26, 1.90)
Model III	1	1.08 (0.88, 1.33)	1.19 (0.96, 1.46)	1.48 (1.19, 1.85)

Model I: crude

Model II: Adjusted for age and sex

Model III: Adjusted for age, sex, BMI, BFM, WHR, carbohydrate (%E), protein (%E) and oil/fat (%E) and physical activity

### Association between dietary inflammatory index and HTN

**Fig. 2:**
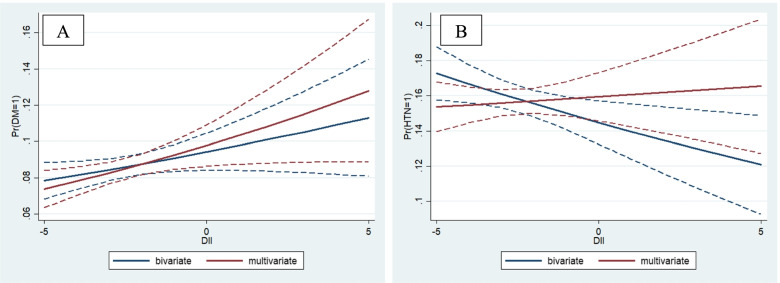
The association between dietary inflammatory index with T2DM (**A**) and hypertension (**B**)

After adjusting for age and gender, the odds of HTN were 0.86 (95% CI: 0.73, 1.0), 0.98 (95% CI: 0.84, 1.15) and 1.06 (95% CI: 0.90, 1.24) across all DII score quartiles, respectively. After adjusting for another confounder in model III, we observed that those in the fourth quartile of DII had an approximately 10% higher odds of HTN than those in the first quartile of DII, although this difference was not statistically significant (Table 4). DII was associated with an increased odd of T2DM and HTN in both the crude and adjusted models (Fig. 2).

**Table 4:** Logistic regression analysis of association between dietary inflammatory index and hypertension

HTN	Dietary Inflammatory Index			
	Q 1 (Most Anti-Inflammatory)	Q 2	Q 3	Q 4 (Most Pro-Inflammatory)
	OR	OR (95% CI)	OR (95% CI)	OR (95% CI)
Model I	1	0.78 (0.67, 0.91)	0.81 (0.69, 0.94)	0.80 (0.69, 0.93)
Model II	1	0.86 (0.73, 1.0)	0.98 (0.84, 1.15)	1.06 (0.90, 1.24)
Model III	1	0.85 (0.73, 1.01)	0.99 (0.84, 1.16)	1.10 (0.92, 1.31)

Model I: crude

Model II: Adjusted for age and sex

Model III: Adjusted for age, sex, BMI, BFM, WHR, carbohydrate (%E), protein (%E) and oil/fat (%E) and physical activity

## Discussion

The current study’s findings demonstrate that the odds of developing T2DM was significantly higher in the fourth quartile of DII (indicating a more pro-inflammatory diet) than in the first quartile of DII (indicating the more anti-inflammatory diet). This association persisted after adjusting for confounding variables such as age, gender, physical activity, and energy intake. According to Laouali et al. (2019), a higher anti-inflammatory diet is associated with a lower risk of T2DM in French adults [23]. Denova-Gutiérrez et al. discovered an association between a pro-inflammatory diet and an increased risk of T2DM in adult Mexicans [24]. We found no correlation between DII and the odds of developing HTN. A cohort study of a large population of French people found a weak correlation between DII and HTN incidence [11]. Simultaneously, an Australian study of women discovered that a pro-inflammatory diet was associated with an increased risk of developing HTN [25]. This discrepancy could be explained by differences in how DII is calculated across studies.

The DII score was calculated in this study using 31 dietary parameters. DII has been calculated in some studies using 27 or 25 dietary parameters [24, 25]. Contradictions in the results are due to differences in the studied populations, genetic factors, and dietary patterns throughout the world, contributing to differences in the DII range.

This study demonstrates that males, urban residents, and married subjects had a significantly higher DII score. Furthermore, the most pro-inflammatory diet resulted in a significantly higher BMI, increased TG, lower SES, decreased physical activity, and decreased HDL-C compared to the most anti-inflammatory diet. These factors are associated with a healthy lifestyle; evidence suggests that lifestyle factors contribute to the prevention or initiation of inflammation. Numerous studies have reported an association between lifestyle changes and anti-inflammatory effects, such that increases in fibre intake and moderate to vigorous leisure-time physical activity predicted decreases in either CRP or IL-6 levels [26, 27]. A longitudinal study conducted in the UK demonstrated a linear relationship between CRP levels and weight gain over 9 years [27]. Moreover, some researchers have suggested that obesity plays a role in the relationship between an anti-inflammatory diet and the odds of developing T2DM or HTN [11, 23]. Our findings indicate that an anti-inflammatory diet is positively associated with a high BMI, highlighting the critical role of lifestyle factors in inflammatory markers.

On the other hand, following a Mediterranean diet low in inflammation has been shown to be a protective factor against T2DM (49% risk reduction) and lower TNF-α, CRP, and IL-6 levels [28]. Single-nutrient studies evaluated the polyphenols in grapes and raisins, both of which have been shown to reduce plasma TNF-α levels [29, 30]. Dietary patterns analysis revealed that subjects who consumed a significant amount of red meat, low-fiber bread and cereals, dried beans, fried potatoes, tomato vegetables, eggs, cheese and drank little wine had a nearly 4.5-fold increased risk of T2DM [31]. Additionally, a significant association between high-salt and high-fat diets and the risk of T2DM was discovered [32]. After 1 year on the Mediterranean diet, CPR decreased by 37%, and adiponectin increased by 43% in a randomized controlled trial (RCT) [33].

Moreover, most high-consumption foods found in the most pro-inflammatory diets, such as red and processed meats, refined grains, and soft drinks, have been linked to elevated inflammatory markers and an increased risk of T2DM [34]. Additionally, CRP levels can be used to predict mortality risk in adults with T2DM [35]. Increased production of the IL-1 family cytokines, IL-1β and IL-18, has been linked to an increased risk of HTN [36, 37]. As a result, it can be concluded that inflammatory markers play a role in developing T2DM and HTN and that diet is a critical factor in increasing or decreasing inflammatory marker levels. The DII is an accurate tool for examining these correlations.

We calculated DII using a validated dietary questionnaire; thus, diet information was collected face-to-face, minimizing measurement error. However, diets may change over time, and we cannot quantify these changes, longitudinal studies examining these associations are required to establish causality. The main limitation of current study was its cross-sectional design, limiting causal inference of the observed associations. The study’s advantages include large sample size and the use of data from a well-designed cohort study, as well as the ability to adjust for potentially confounding variables. Additionally, the first study is based on a sizable population of Kurdish subjects, allowing for comparisons with other ethnic groups worldwide. The results of this study can be generalized to adults of Kurdish ethnicity because of the similarity of diet in the Kurdish ethnic groups of Iran and neighboring countries.

## Conclusion

In conclusion, we found a significant association between a pro-inflammatory diet and the odds of developing T2DM, but only a weak association between a pro-inflammatory diet and HTN. The subjects who consumed the most pro-inflammatory diet had a significantly higher BMI, TG, lower SES, physical activity, and HDL-C than those who consumed a primarily anti-inflammatory diet. As a result of the current study’s findings, an anti-inflammatory diet may help prevent the development of HTN, T2DM, and their associated risk factors. Modification of diet is recommended to reduce inflammation.

## Data Availability

The data sets generated during this study are available from the correspondence author on reasonable request via email.

## Abbreviations

NCDs: Non-communicable diseases
CVDs: Cardiovascular disease
T2DM: Type 2 diabetes mellitus
MetS: Metabolic syndrome
CRP: C-reactive protein
DII: Dietary inflammatory index
RaNCD: Ravansar Non-Communicable Diseases
FFQ: Food frequency questionnaire
SES: Socio-economic status
PERSIAN: Prospective Epidemiological Research Studies in IrAN
PCA: Principal component analysis
TG: Triglyceride
LDL-C: Low-density lipoprotein Cholesterol
HDL-C: High-density lipoprotein Cholesterol
T-C: Total cholesterol
FBS: Fasting blood sugar
BMI: Body mass index
BFM: Body fat mass
VFA: visceral fat area
WC: Waist circumference
WHR: Waist to hip ratio
BP: Blood pressure
DBP: Diastolic Blood Pressure
SBP: Systolic Blood Pressure
Il-*1β*: *Interleukin-1β*
Il-4: Interleukin 4
Il-6: Interleukin 6
Il-10: Interleukin 10
MUFA: Monounsaturated fatty acids
PUFA: Polyunsaturated fatty acids
RCT: Randomized controlled trial
CI: Confidence interval
SD: Standard deviation
